# Microwave-Promoted Total Synthesis of Puniceloid D for Modulating the Liver X Receptor

**DOI:** 10.3390/molecules29020416

**Published:** 2024-01-15

**Authors:** Young Jin Jung, Narges Hosseininasab, Jungjin Park, Soonsil Hyun, Jae-Kyung Jung, Jae-Hwan Kwak

**Affiliations:** 1 College of Pharmacy, Kyungsung University, Busan 48434, Republic of Korea; louisj0750@naver.com; 2 College of Pharmacy, Chungbuk National University, Cheongju 28160, Republic of Korea; nargeshosseini@chungbuk.ac.kr (N.H.); jjpark0823@chungbuk.ac.kr (J.P.); shyun@chungbuk.ac.kr (S.H.); orgjkjung@chungbuk.ac.kr (J.-K.J.)

**Keywords:** liver X receptors, nuclear receptor, puniceloid D, total synthesis, natural products

## Abstract

A growing global health concern is metabolic syndrome, which is defined by low HDL, diabetes, hypertension, and abdominal obesity. Nuclear receptors are attractive targets for treatment of diseases associated with metabolic syndrome. Liver X receptors (LXRs) have become one of the most significant pharmacological targets among nuclear receptors. Multiple research studies emphasize the essential function of the liver X receptor (LXR) in the pathophysiology of metabolic syndrome. Puniceloid D, among natural products, demonstrated promising effects on LXRα. However, attempts at the total synthesis of natural products were faced with challenges, including long synthetic steps and low yields, requiring a more efficient approach. In this study, for the first time, we successfully synthesized puniceloid D through a seven-step process and conducted docking studies to gain a comprehensive understanding of the interactions involved in the binding of puniceloid D to LXR within different heterodimeric contexts. Our understanding of the pathophysiology of metabolic syndrome could be improved by these findings, which might assist with the development of novel treatment strategies.

## 1. Introduction

One of the major obstacles for health systems worldwide is the diseases associated with metabolic syndrome. The condition consists of several risk factors, particularly linked to diabetes and cardiovascular disease. The cluster of metabolic factors includes low HDL cholesterol, high triglyceride levels, impaired fasting glucose, high blood pressure, and abdominal obesity [1]. Therefore, the management and treatment of metabolic syndrome are of highest priority. Recent study data indicate that nuclear receptors play a critical role in the pathophysiology of this condition [2].

Target gene expression in a wide range of physiological pathways, including metabolic processes, is regulated by nuclear receptors, which are ligand-activated transcription factors [3,4,5]. Nuclear receptors associated with metabolism are categorized as type 2 nuclear receptors, which are found inside the nucleus regardless of ligand binding [6]. Liver X receptors (LXRα (NR1H3) and LXRβ (NR1H2)), farnesoid X receptor (FXR, NR1H4), peroxisome proliferator-activated receptors (PPARs, NR1C1/NR1C2/NR1C3), and RXRs are examples of type 2 nuclear receptors for the regulation of metabolic processes. Fatty acids, oxysterols, bile acids, and rexinoids are ligands for these receptors, highlighting their importance in the control of metabolic pathways [7,8].

An essential role for liver X receptors is to regulate innate immunity, inflammatory responses, and lipid and cholesterol metabolism [9,10,11]. There are two isoforms of the liver X receptor: LXRα and LXRβ [12]. While LXRβ (NR1H2) is expressed everywhere, LXRα (NR1H3) is expressed in metabolically active tissues such as the liver, adipose, kidney, macrophages, and intestines [13,14]. LXR inhibits gluconeogenesis, promotes bile secretion, raises cholesterol clearance in the liver, suppresses the macrophage inflammatory response, increases clearance of cholesterol in the small intestine, and facilitates glucose reabsorption into cells by promoting the expression of GLUT4 (glucose transporter type 4) in adipose tissue [15]. In addition to their role in regulating lipid metabolism, LXRs play a crucial role in controlling blood sugar levels by modulating glucose transport proteins such as GLUT4/5, and regulatory proteins like ChREBP (carbohydrate responsive element-binding protein), which are involved in glucose homeostasis [16]. Therefore, the majority of the research performed to identify LXR modulators with therapeutic potential has focused on the development of LXR agonists [17,18].

**Figure 1:**
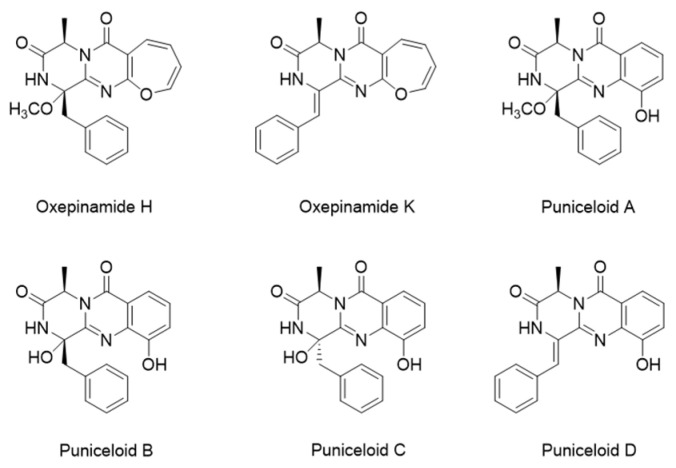
Structures of natural product-oriented LXRα agonists.

The search for LXR modulators for the treatment of metabolic diseases is ongoing. Utilizing natural products that potentially offer decreased potency and fewer side effects or demonstrate tissue- or subtype-selective activity represents a viable approach [1]. Figure 1 displays some discovered LXRα agonists derived from natural products (marine-derived fungi) [19]. Based on a study by Liang et al., [19] puniceloid C and D were identified as the most potent LXRα agonists.

Quinazolinones are known for their diverse and significant biological properties, including cholinesterase inhibition and antitumor, antiviral, anti-inflammatory, anticancer, and protein kinase inhibitory effects [20]. The presence of quinazolinone and its derivatives is noted in over 100 naturally occurring alkaloids, comprising a crucial class of fused heterocycles [21]. Several families of alkaloids are represented by fungal quinazolinone metabolites that contain the core pyrazino[2,1-*b*]quinazoline-3,6-dione (**A**) scaffold [22].

**Figure 2:**
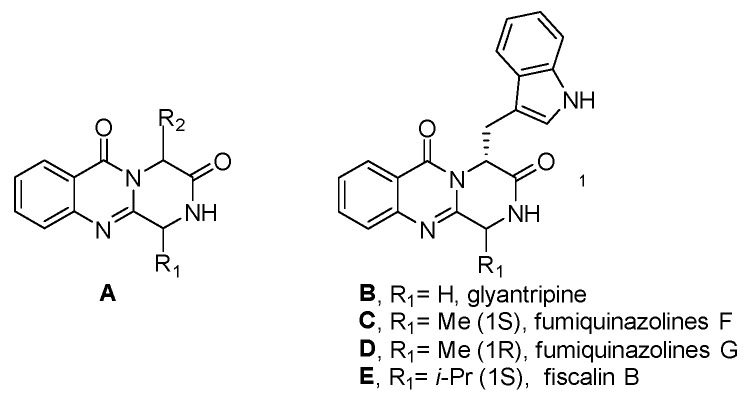
Natural products containing pyrazino[2,1-*b*]quinazoline-3,6-dione (**A**).

Scaffold **A** is a structural motif found in several natural products known for their notable biological activities, such as glyantripine (**B**), fiscalin B (**C**), fumiquinazolines F (**D**) and G (**E**), as well as puniceloids A−D (Figure 2).

**Figure 3:**
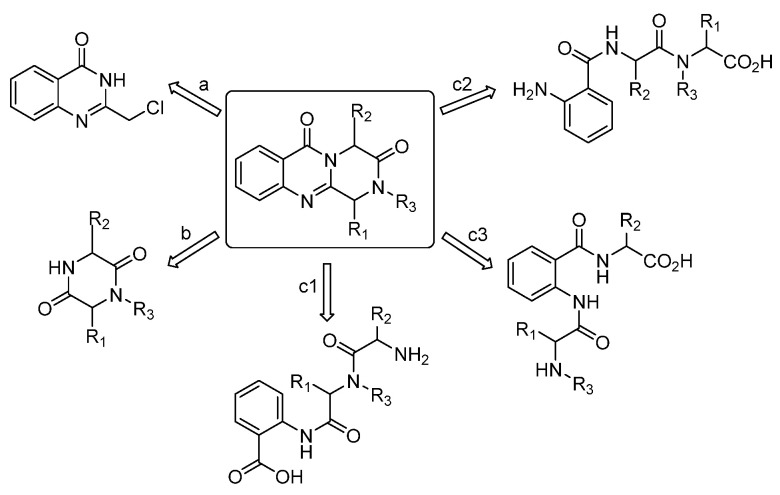
Different synthetic approaches to the parent ring system (pyrazino[2,1-*b*]quinazoline-3,6-dione).

Derivatives of this system have been synthesized using three distinct methods: the cyclization of 4(3*H*)-quinazolinones (a), the cyclocondensation of 2,5-piperazinediones and anthranilic acid derivatives (b), and the use of open-chain tripeptides, with anthranilic acid positioned at the C-terminal (c1), N-terminal (c2), or as the intermediate residue (c3) [23] (Figure 3).

Considering the increasing interest in natural products as potential sources of novel drugs [24] or as lead structures for drug discovery in medicinal and organic chemistry [25,26], and drawing inspiration from existing data [19] highlighting puniceloid D as the most potent LXRα agonist, our study presents the total synthesis of puniceloid D for the first time. Furthermore, significant results from molecular docking studies were obtained with puniceloid D.

## 2. Results

### Total Synthesis of Puniceloid D

**Scheme 1:**
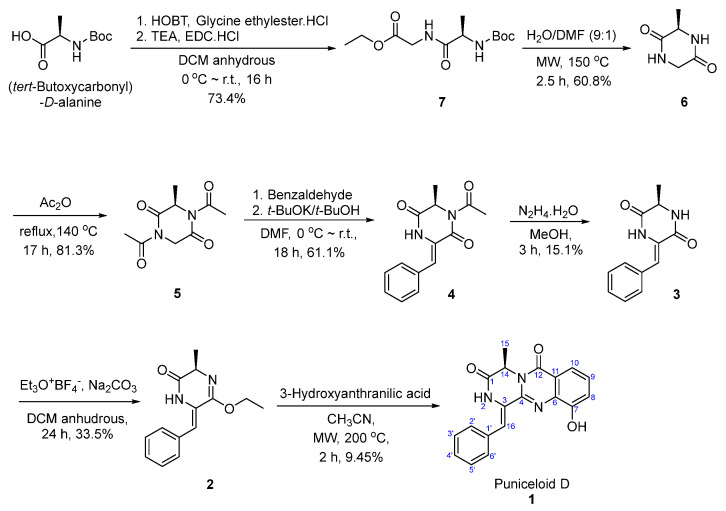
Total synthesis of puniceloid D (**1**).

The first total synthesis of puniceloid D (**1**) involved seven steps. In our study, we chose route (b) (Figure 3) based on literature findings, as protecting N-2 was reported to enhance reactivity and prevent racemization [27]. In the first step, the reaction of (*tert*-butoxycarbonyl)-*D*-alanine with glycine ethyl ester hydrochloride as a nucleophile produced ethyl (*tert*-butoxycarbonyl)-*D*-alanylglycinate (**7**) at a 73.4% yield. This reaction was facilitated by using EDC and HOBt, which convert amine to amide [28]. According to the literature, the stereochemistry is preserved [29,30]. In the second step, intramolecular cyclization occurred under microwave conditions, involving the removal of the Boc group. Subsequently, intramolecular nucleophilic addition of the amin in compound **7** to the carbonyl group, followed by the elimination of the ethoxy group, produced compound **6** [31]. The literature states that compound **6** was obtained with complete retention of the original stereochemical configuration of **7** through the use of HPLC analysis on the chiral phase [31]. Next, compound **6** was heated under reflux conditions in acetic anhydride to yield compound **5**. Classically, an excess of acetic anhydride is used in solvent-free acetylation reactions and it preserves the stereochemistry [32]. To facilitate base-catalyzed condensation with aldehydes, compound **5** was diacetylated in the previous step. At room temperature, an aldol reaction is carried out between compound **5** and benzaldehyde, in the presence of potassium *t*-butoxide and DMF as a solvent, to form compound **4** [27]. The acetyl group reduces the pKa of the alpha carbonyl, making it easily deprotonated. Based on existing research, compound **4** maintains its stereochemical integrity [27,33]. Challenges in E/Z isomer selectivity could arise depending on the location of the phenyl ring. However, in the literature, the absence of NOE enhancement in vinyl protons upon N-H signal irradiation confirmed the *Z* configuration [27,34]. Compound **4** was deacetylated through treatment with hydrazine monohydrate, resulting in the formation of compound **3** [27]. The removal of the acetyl group increased the polarity of the compound, making it less soluble in commonly used solvents such as ethyl acetate and dichloromethane. The reaction of compound **3** with Meerwein’s salt (triethyloxonium tetrafluoroborate) in the presence of sodium carbonate resulted in the formation of the monoiminoether compound (**2**) [28,31]. Meerwein’s salt can act as an alkylating agent by serving as a source of ethyl groups (C_2_H_5_^+^) [35]. In accordance with published research [36], thermal treatment of compound **3** does not cause any changes to the stereocenter, leading to compound **2**. Despite the reaction running for 24 h, the yield was low because the starting material for this reaction was diketopiperazine, which has low reactivity. The number of equivalents of the Meerwein’s salt was increased to enhance the yield, but when 2.2 equivalents were used, it was discovered that ethylation occurred on both carbonyl oxygens in the diketopiperazine ring, highlighting the importance of controlling the number of equivalents. Finally, compound **2** interacts with 3-hydroxyanthranilic acid under microwave conditions at 200 °C, followed by cyclization and elimination of hydroxy, resulting in the formation of puniceloid D (**1**) [37] (Scheme 1). In the literature, certain research groups have focused on producing 1-aryl methylene pyrazino[2,1-*b*]quinazoline-3,6-diones, which share similarities with puniceloid D. Cledera et al. detailed a seven-step synthesis of (*R*,*Z*)-1-(4-methoxybenzylidene)-4-methyl-1,2-dihydro-6*H*-pyrazino[2,1-*b*]quinazoline-3,6(4*H*)-dione under heating conditions at 120 °C for 2 h, achieving a 15% yield [27]. The same group also employed a 9-min microwave irradiation reaction, obtaining the same compound at a 26% yield [36]. Taking inspiration from this, we successfully synthesized puniceloid D (**1**) for the first time with a 9.45% yield, utilizing 3-hydroxyanthranilic acid under microwave irradiation for 2 h.

The ^1^H NMR and ^13^C NMR spectra of the final synthesized compound (**1**) exhibited a complete match with the reported ones [19] (Table 1) (Figure S15).

## 3. Discussion

According to the literature [19], the transactivation effects of puniceloid D and oxepinamide K on LXRα were investigated. Puniceloid D exhibited significant transcriptional activation on LXRα, demonstrating an EC_50_ value of 1.7 μM. In contrast, oxepinamide K, which contains an oxepin unit, showed 29 times less active transcriptional activation on LXRα, with EC_50_ values of 50 μM compared to puniceloid D. These findings indicate that the quinazolinone skeleton found in puniceloid D is essential in determining the observed bioactivity in the context of LXRα transactivation [38]. To enhance our understanding of the binding modes of puniceloid D and oxepinamide K with LXRs, we conducted molecular docking studies (in silico) using Glide in Extra Precision (XP), as implemented in Schrödinger (version 2023-4). The docking studies were performed with LXRα in the context of two different LXRα–RXRβ and LXRα–RXRα LBD heterodimers, represented by PDB IDs 1UHL and 2ACL, respectively.

**Figure 4:**
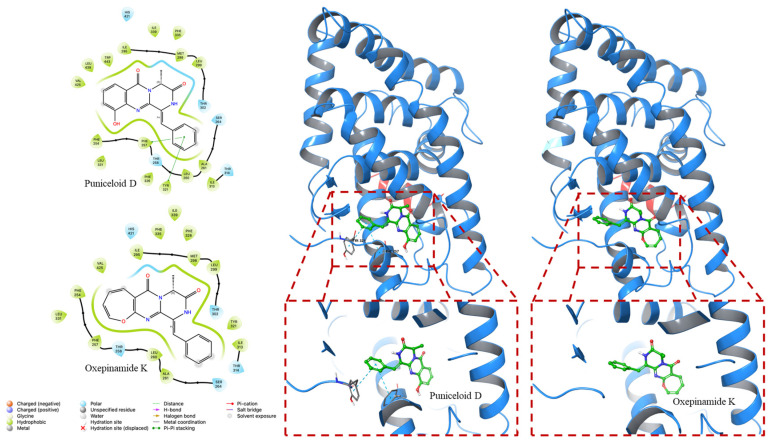
In silico docking study of puniceloid D and oxepinamide K with LXRα of LXRα–RXRβ LBD heterodimer (PDB ID: 1UHL). Puniceloid D exhibited π–π stacking interactions with Phe257 and Tyr32.

**Figure 5:**
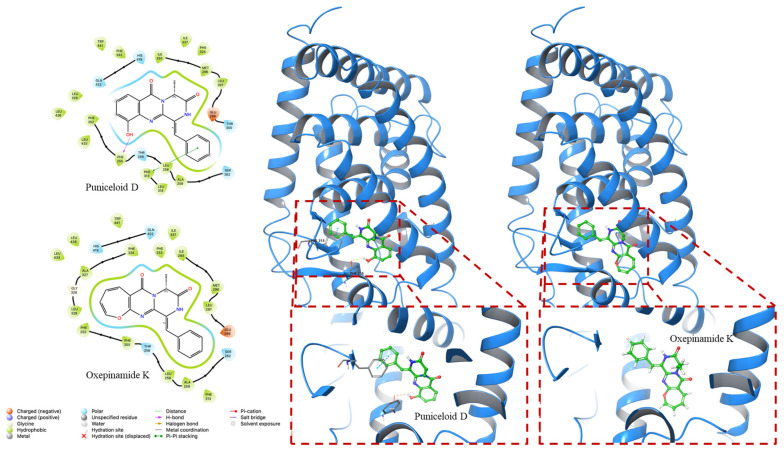
In silico docking study of puniceloid D and oxepinamide K with LXRα of LXRα–RXRα LBD heterodimer (PDB ID: 2ACL). Puniceloid D exhibited π–π stacking interactions with Phe313 and hydrogen bonding interactions with Phe255.

In our in silico docking study, we investigated the interactions of puniceloid D and oxepinamide K with two LXRα LBD (PDB ID 1UHL and PDB ID 2ACL). Puniceloid D notably exhibited π–π stacking interactions with Tyr32 and Phe257 when docked with LXRα from the LXRα–RXRβ heterodimer (Figure 4). Similarly, in the docking study with LXRα from the LXRα–RXRα heterodimer, puniceloid D displayed π–π stacking interactions with Phe313 and hydrogen bonding interactions with Phe255 (Figure 5). This revealed different binding modes and potential molecular mechanisms underlying its interactions with LXRα in various heterodimeric contexts. Furthermore, we used the “dock” mode to calculate the binding affinity of compounds based on minimum energy values (kcal/mol) (Table 2). In both PDB IDs (1UHL and 2ACL), puniceloid D exhibited the lowest docking scores (−8.535 and −9.783 kcal/mol, respectively) and G scores (−8.841 and −10.098 kcal/mol, respectively) compared to oxepinamide K, indicating more stable binding conformations. This work provides more evidence that the quinazolinone skeleton is a useful model for the synthesis of LXR agonists.

## 4. Materials and Methods

### 4.1. Materials and Synthetic Methods

NMR spectra were acquired using Jeol JNM-ECX400 spectrometers (Jeol, Tokyo, Japan) operating at 400 MHz for ^1^H and at 100 MHz for ^13^C. Chemical shifts were reported in parts per million (ppm), and coupling constants were expressed in hertz (Hz). High-resolution mass spectra (HRMS) were obtained in positive mode using electrospray ionization (ESI-MS) with an Agilent 6530 accurate-mass quadrupole time-of-flight (Q-TOF) mass spectrometer (Agilent, Santa Clara, CA, USA). Biotage Isolera^TM^ (Uppsala, Sweden) was used for medium-pressure liquid chromatography (MPLC), and silica gel (ZEOprep 60, 40–63 μm, ZEOCHEM, Rüti, Switzerland) was utilized for column chromatography. A Kieselgel 60 F254 plate from Merck (Darmstadt, Germany) was used for thin-layer chromatography (TLC). The reagents and solvents utilized in this experiment were commercially available and used without any additional purification steps.

#### 4.1.1. Synthesis of Ethyl (*tert*-Butoxycarbonyl)-*D*-alanylglycinate (**7**)

(*tert*-Butoxycarbonyl)-*D*-alanine (1.0 g, 5.3 mmol) was dissolved in anhydrous dichloromethane (DCM) (38 mL) in an ice bath. To the dissolved solution, 1-hydroxybenzotriazole (HOBt) (1.07 g, 7.93 mmol) and glycine ethyl ester hydrochloride (1.48 g, 10.6 mmol) were added. After 5 min, triethylamine (TEA) (3.32 mL, 23.8 mmol) was added dropwise, followed by the addition of 1-ethyl-3-[3-(dimethylamino)propyl]carbodiimide hydrochloride (EDC.HCl) (1524 mg, 7.93 mmol). The ice bath was then removed, and the reaction mixture was stirred at room temperature for 16 h. Upon completion of the reaction, the solution was diluted with DCM and washed with 1N HCl. Then, the combined organic layers were washed with a saturated NaHCO_3_ solution. The aqueous layer was further extracted twice with DCM. Moisture was removed by passing the organic layer through anhydrous MgSO_4_ and filtering. Purification was carried out using medium-pressure liquid chromatography (MPLC) with an elution solvent mixture of ethyl acetate and hexane in a ratio of 2:3, and compound **7** was obtained with a yield of 73.4%. Figure S1: ^1^H NMR (400 MHz, CDCl_3_) *δ* 6.63 (brs, 1H), 4.97 (brs, 1H), 4.21 (q, *J* = 7.1 Hz, 3H), 4.03 (m, 2H), 1.45 (s, 9H), 1.38 (d, *J* = 7.4 Hz, 3H), 1.28 (t, *J* = 7.3Hz, 3H) [30]. Figure S2: ^13^C NMR (100 MHz, CD_3_OD) *δ* 176.4, 171.1, 157.6, 80.6, 62.2, 51.5, 42.0, 28.6, 18.4, 14.4.

#### 4.1.2. Synthesis of (*R*)-3-methylpiperazine-2,5-dione (**6**)

Ethyl (*tert*-butoxycarbonyl)-*D*-alanylglycinate (**7**) (1.07 g, 3.89 mmol) was dissolved in dimethylformamide (DMF) (7.8 mL). Subsequently, 70.2 mL of water was added. The reaction solution was heated at 150 °C for 2 h and 30 min using a microwave. After completion of the reaction, methanol was added to the reaction mixture, and the solvent was concentrated using a rotary evaporator. The resulting material was then subjected to purification through silica gel filtration under the conditions of DCM and MeOH in a ratio of 95:5, and compound **6** was obtained with a yield of 60.8%. Figure S3: ^1^H NMR (400 MHz, CD_3_OD) *δ* 4.00 (q, *J* = 7.0 Hz, 1H), 3.90 (m, 2H), 1.41 (d, *J* = 7.0 Hz, 3H) [31]. Figure S4: ^13^C NMR (100 MHz, CD_3_OD) *δ* 171.6, 168.7, 51.6, 45.4, 19.4.

#### 4.1.3. Synthesis of (*R*)-1,4-diacetyl-3-methylpiperazine-2,5-dione (**5**)

(*R*)-3-Methylpiperazine-2,5-dione (**6**) (303 mg, 2.36 mmol) and acetic anhydride (6.26 mL, 0.38 M) were combined and refluxed at 130 °C for 17 h. After completion of the reaction, acetic anhydride was removed by evaporating through a rotary evaporator, and the concentrated solution was purified using MPLC with an elution solvent mixture of ethyl acetate and hexane in a ratio of 1:3. Compound **5** was obtained with a yield of 81.3%. Figure S5: ^1^H NMR (400 MHz, CD_3_OD) *δ* 5.10 (q, *J* = 7.3 Hz, 1H), 4.20–4.96 (q, *J* = 18 Hz, 2H), 2.48 (s, 3H), 2.47 (s, 3H), 1.47 (d, *J* = 7.3 Hz, 3H) [39]. Figure S6: ^13^C NMR (100 MHz, CD_3_OD) *δ* 172.8, 172.6, 170.4, 167.6, 55.4, 47.3, 27.0, 26.9, 17.6.

#### 4.1.4. Synthesis of (*R*,*Z*)-1-acetyl-3-benzylidene-6-methylpiperazine-2,5-dione (**4**)

(*R*)-1,4-Diacetyl-3-methylpiperazine-2,5-dione (**5**) (405 mg, 1.92 mmol) was dissolved in dimethylformamide at 0 °C (5.05 mL), followed by the dropwise addition of benzaldehyde (0.502 mL, 2.56 eq). Subsequently, a 1 M solution of *t*-BuOK/*t*-BuOH (2.30 mL, 1.2 eq) was slowly added dropwise. After 5 min, the ice bath was removed, and the reaction mixture was stirred at room temperature for 18 h. Upon completion of the reaction, a saturated NH_4_Cl solution was added to quench the reaction, followed by extraction three times with ethyl acetate. Moisture was removed using anhydrous MgSO_4_, and the solvent evaporated. The resulting material was purified under the conditions of ethyl acetate and hexane in a ratio of 1:3. Compound **4** was obtained with a yield of 61.1%. Figure S7: ^1^H NMR (400 MHz, CDCl_3_) *δ* 7.85 (brs, 1H), 7.49–7.37 (m, 5H), 7.18 (s, 1H), 5.16 (q, *J* = 7.1 Hz, 1H), 2.61 (s, 3H), 1.57 (d, *J* = 7.1 Hz, 3H) [33]. Figure S8: ^13^C NMR (100 MHz, CDCl_3_) *δ* 172.0, 167.1, 160.9, 132.6, 129.6, 129.6, 128.8, 125.7, 120.3, 52.8, 27.1, 20.0.

#### 4.1.5. Synthesis of (*R*,*Z*)-3-benzylidene-6-methylpiperazine-2,5-dione (**3**)

(*R*,*Z*)-1-Acetyl-3-benzylidene-6-methylpiperazine-2,5-dione (**4**) (95.0 mg, 0.368 mmol) was dissolved in anhydrous methanol (6.69 mL). Hydrazine hydrate (0.0386 mL) was then added dropwise to the reaction mixture, resulting in the formation of a solid in the reaction solution. After the completion of the reaction, the product was purified through recrystallization, and compound **3** was obtained with a yield of 15.1%. Figure S9: ^1^H NMR (400 MHz, DMSO-*d_6_*) δ = 9.89 (brs, 1H), 8.45 (brs, 1H), 7.48 (d, *J* = 7.14 Hz, 2H), 7.38 (t, *J* = 7.86 Hz, 2H), 7.28 (t, *J* = 7. 37 Hz, 1H), 6.81 (s, 1H), 4.10 (q, *J* = 6.9 Hz, 1H), 1.31 (d, *J* = 7.0 Hz, 3H) [40,41]. Figure S10: ^13^C NMR (100 MHz, DMSO-*d_6_*) *δ* 167.7, 160.3, 133.4, 129.2, 128.6, 127.9, 127.0, 114.0, 50.3, 19.3.

#### 4.1.6. Synthesis of (*R*,*Z*)-6-benzylidene-5-ethoxy-3-methyl-3,6-dihydropyrazin-2(1*H*)-one (**2**)

(*R*,*Z*)-3-Benzylidene-6-methylpiperazine-2,5-dione (**3**) (82.0 mg, 0.379 mmol) and Na_2_CO_3_ (241 mg, 2.27 mmol) were dissolved in anhydrous DCM (5.41 mL). Meerwein’s salt (1 M, 1.2 eq)—dissolved in DCM—was then added, and the mixture was stirred at room temperature for 24 h. After the reaction was complete, ice was placed into the reaction vessel, and the resulting mixture was extracted three times with DCM. Moisture was removed through anhydrous MgSO_4_, followed by filtration and evaporation. Purification was carried out through MPLC under the conditions of ethyl acetate and hexane in a ratio of 1:4. Compound **2** was obtained with a yield of 33.5%. Figure S11: ^1^H NMR (400 MHz, CDCl_3_) δ = 7.75 (s, 1H), 7.40 (d, *J* = 7.24 Hz, 2H), 7.33 (d, *J* = 6.84 Hz, 2H), 7.32 (m, 1H), 6.54 (s, 1H), 4.39 (q, *J* = 7.25 Hz, 1H), 4.27 (m, 2H), 1.55 (d, *J* = 7.27 Hz, 3H), 1.39 (t, *J* = 7.0 Hz, 3H). Figure S12: ^13^C NMR (100 MHz, CDCl_3_) *δ* 173.1, 155.4, 134.7, 130.0, 129.8, 129.1, 124.5, 112.6, 63.2, 57.0, 21.5, 14.5.

#### 4.1.7. Synthesis of Puniceloid D (**1**)

(*R*,*Z*)-6-Benzylidene-5-ethoxy-3-methyl-3,6-dihydropyrazin-2(1*H*)-one (**2**) (31.0 mg, 0.127 mmol) was dissolved in anhydrous acetonitrile (1.49 mL). Subsequently, 3-hydroxyanthranilic acid (25.3 mg, 0.165 mmol) was added, and the reaction was carried out at 200 °C for 2 h under microwave conditions. After evaporating the solvent, the product was purified using ethyl acetate and hexane in a ratio of 1:2. The target compound (**1**) was obtained with a yield of 9.45%. Figure S13: ^1^H NMR (400 MHz, DMSO-*d_6_*) *δ* 10.56 (s, 1H), 9.76 (s, 1H), 7.71 (s, 1H), 7.66 (d, *J* = 7.71 Hz, 2H), 7.58 (dd, *J* = 1.4, 7.9 Hz, 1H), 7.47 (t, *J* = 7.62 Hz, 2H), 7.40–7.37 (m, 1H), 7.37 (t, *J* = 7.9 Hz, 1H), 7.26 (dd, *J* = 1,4, 7.9 Hz, 2H), 5.22 (q, *J* = 6.8 Hz, 1H), 1.49 (d, *J* = 7.0Hz, 3H). Figure S14: ^13^C NMR (100 MHz, CDCl_3_) *δ* 166.3, 160.0, 151.7, 143.3, 135.6, 133.0, 129.7, 129.2, 128.6, 128.7, 125.9, 120.4, 117.8, 117.7, 116.3, 52.0, 19.5. HRMS (ESI, m/z): [M + H]^+^ calculated for C_19_H_15_N_3_O_3_, 334.1191; found 334.1189.

### 4.2. Molecular Docking Experiment

The docking studies were conducted using Glide in Extra Precision (XP) employed by Schrödinger (version 2023-4). LXRα structures were acquired from the Protein Data Bank (PDB) with codes 1UHL and 2ACL [42]. The 2D structures of the compounds were obtained in .sdf file format using CS ChemDraw (version 20) and were subsequently converted into 3D structures. The ligands were independently docked into the energy-minimized structures of the ligand-binding domains (LBD) of LXRα. Docking scores and G scores were calculated to assess the binding of each tested compound with LXRα.

## 5. Conclusions

Diseases related to metabolic syndrome, such as obesity and diabetes, exhibit a concerning global prevalence, making them significant health priorities. The LXRs play a major role in regulating lipid and cholesterol metabolism, in addition to their anti-inflammatory activities. Natural products can act as both independent sources for novel drugs and foundational structures for drug discovery. In the present study, we pursued a seven-step synthetic pathway, starting from readily available and inexpensive starting materials, to accomplish the synthesis of the natural product puniceloid D—a novel and potent liver X receptor agonist. Furthermore, the molecular docking study revealed that puniceloid D exhibited favorable binding scores with two LXRα LBD structures (PDB ID: 1UHL and 2ACL) in comparison to oxepinamide K. Puniceloid D demonstrated a favorable conformational state, engaging in hydrogen bond and π–π stacking interactions within the active sites of LXRα. Promising natural products can be chemically modified to overcome their low yield and complicated synthetic pathways, thereby increasing their commercial potential. Our future research will explore the chemical modification of puniceloid D and investigating the mechanisms of action of its compounds would also be of interest.

## Figures and Tables

**Table 1:** Comparative NMR (^1^H (400 MHz) and ^13^C (100 MHz)) analysis of the synthesized puniceloid D (**1**) and the reported results.

Position	Synthesized		Reported [19]	
	δ_C_, Type	δ_H_ (*J* in Hz)	δ_C_, Type	δ_H_ (*J* in Hz)
1	166.3	data	166.6, C	
2-NH		10.56, s		10.52, s
3	125.9		126.0, C	
4	143.3		144.1, C	
6	135.6		136.1, C	
7	151.7		152.8, C	
7-OH		9.76, s		9.75, s
8	117.8	7.26, dd (7.9, 1.4)	118.4, CH	7.26, dd (8.0, 1.0)
9	128.6	7.37, t (7.9)	128.1, CH	7.35, t (8.0)
10	116.3	7.58, dd (7.9, 1.4)	116.0, CH	7.58, dd (8.0, 1.0)
11	120.4		120.4, C	
12	160.0		159.5, C	
14	52.0	5.22, q (6.8)	51.2, CH	5.22, q (7.0)
15	19.5	1.49, d (7.0)	18.2, CH3	1.50, d (7.0)
16	117.7	7.71, s	117.2, CH	7.74, s
1′	133.0		134.0, C	
2′,6′	129.2	7.66, d (7.71)	129.4, CH	7.65, d (7.5)
3′,5′	128.7	7.47, t (7.62)	128.7, CH	7.47, t (7.5)
4′	129.7	7.37, m	127.6, CH	7.37, m

**Table 2:** Glide XP docking scores for puniceloid D and oxepinamide K with two LXRα LBD (PDB ID: 1UHL and 2ACL).

PDB ID	Compounds	Docking Score (Kcal/mol)	G Score (Kcal/mol)
1UHL	Puniceloid D	−8.535	−8.841
	Oxepinamide K	−8.113	−8.113
2ACL	Puniceloid D	−9.783	−10.089
	Oxepinamide K	−8.252	−8.252

## Data Availability

All the data were provided in the main manuscript and Supplementary Materials.

## Supplementary Materials

The following supporting information can be downloaded at: https://www.mdpi.com/article/10.3390/molecules29020416/s1, Figures S1–S14: ^1^H NMR and ^13^C NMR of compounds **1**–**7**; Figure S15: Comparative NMR Data of **1** (A) and the reported one (B).
