# Supplementary material for: Microwave-Promoted Total Synthesis of Puniceloid D for Modulating the Liver X Receptor
Source: Molecules. 2024 Jan 15;29(2):416. doi: 10.3390/molecules29020416 (PMC10821398; doi:10.3390/molecules29020416)
Supplement: Supplementary file 1 [file molecules-29-00416-s001.zip › molecules-2807108-supplementary.pdf]

Microwave-Promoted Total Synthesis of Puniceloid D for Modulating the Liver X Receptor

Young Jin Jung [1,†], Narges Hosseininasab [2,†], Jungjin Park [2], Soonsil Hyun [2], Jae-Kyung Jung [2] and Jae-Hwan Kwak [1,2,*]

[1] College of Pharmacy, Kyungsung University, Busan, 48434, Republic of Korea; louisj0750@naver.com
[2] College of Pharmacy, Chungbuk National University, Cheongju, 28160, Republic of Korea; nargeshosseini@chungbuk.ac.kr, jjpark0823@chungbuk.ac.kr, shyun@chungbuk.ac.kr, orgjkjung@chungbuk.ac.kr
* Correspondence: jhkwak@chungbuk.ac.kr; Tel.: +82-43-261-2997
† These authors contributed equally to this work.

Supporting information

Supporting Figure captions:

Figure S1: ^{1}H NMR Spectrum of 7

Figure S2: ^{13}C NMR Spectrum of 7

Figure S3: ^{1}H NMR Spectrum of 6

Figure S4: ^{13}C NMR Spectrum of 6

Figure S5: ^{1}H NMR Spectrum of 5

Figure S6: ^{13}C NMR Spectrum of 5

Figure S7: ^{1}H NMR Spectrum of 4

Figure S8: ^{13}C NMR Spectrum of 4

Figure S9: ^{1}H NMR Spectrum of 3

Figure S10: ^{13}C NMR Spectrum of 3

Figure S11: ^{1}H NMR Spectrum of 2

Figure S12: ^{13}C NMR Spectrum of 2

Figure S13: ^{1}H NMR Spectrum of 1

Figure S14: ^{13}C NMR Spectrum of 1

Figure S15: Comparative NMR Data of 1 (A) and the reported one (B)

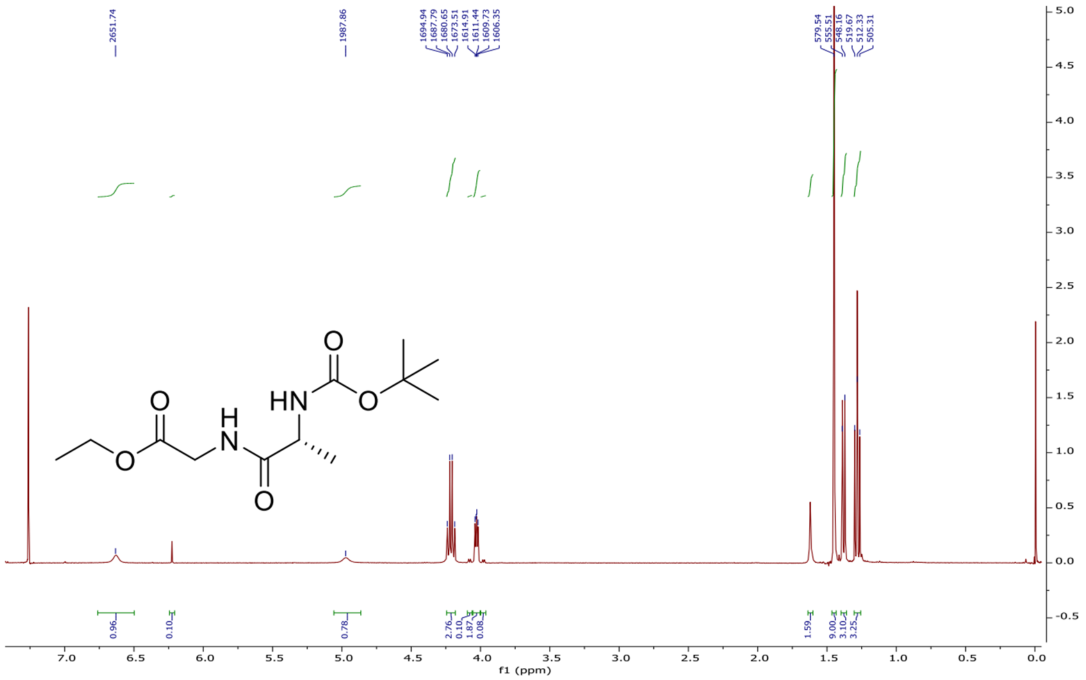

Figure S1

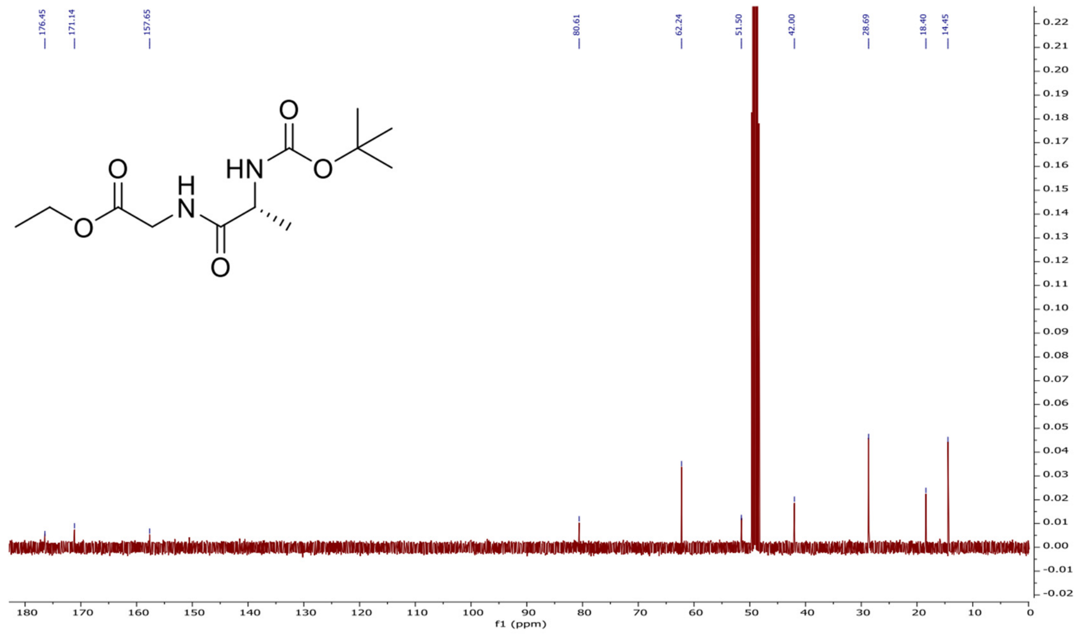

Figure S2

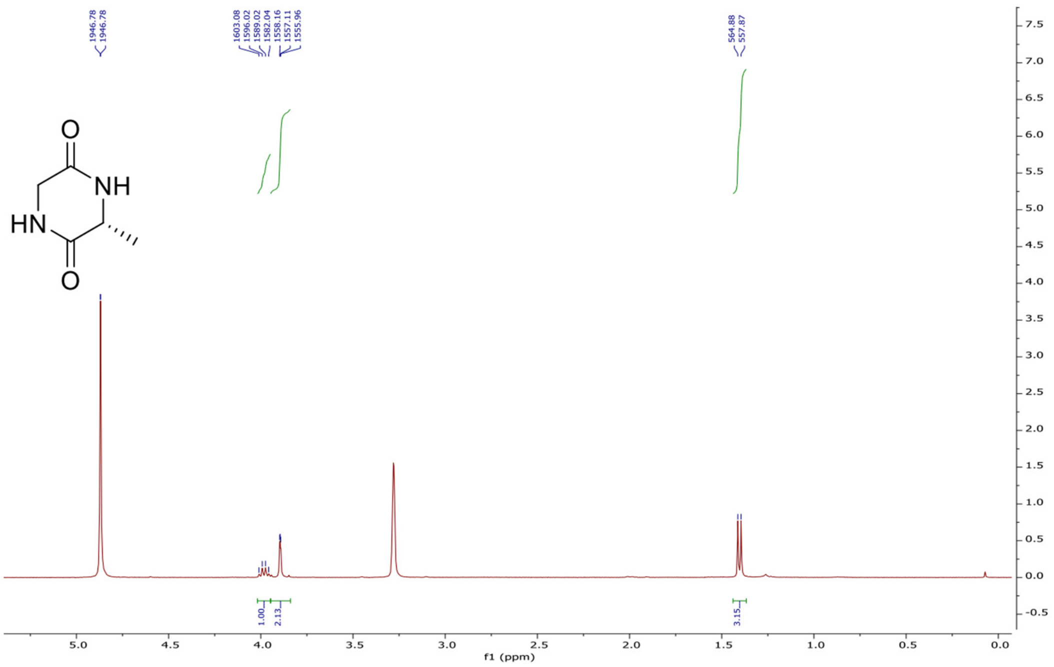

Figure S3

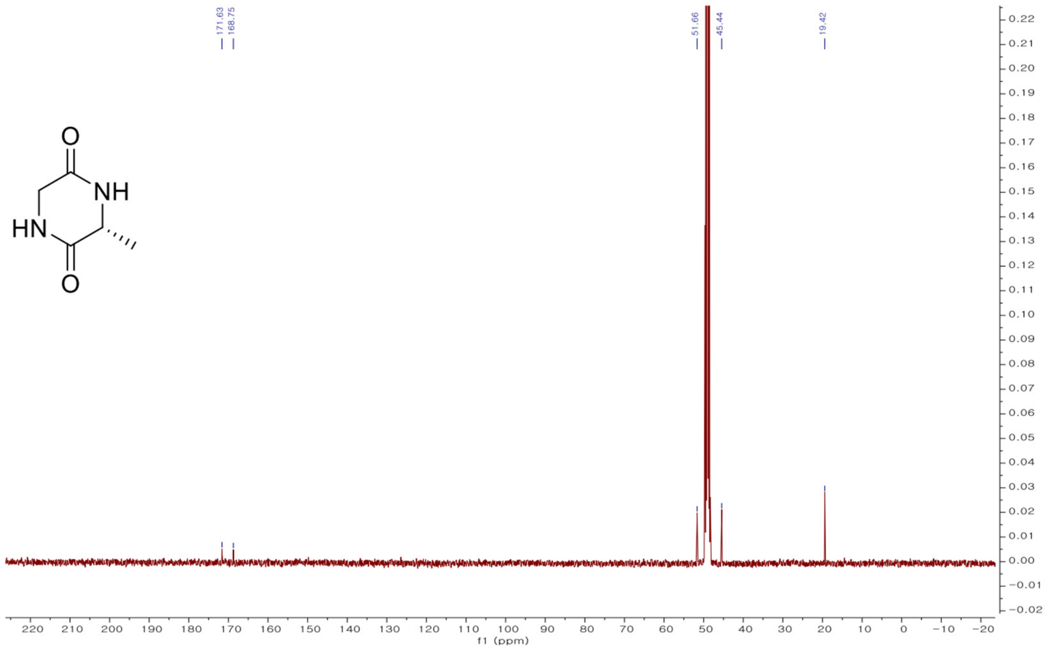

Figure S4

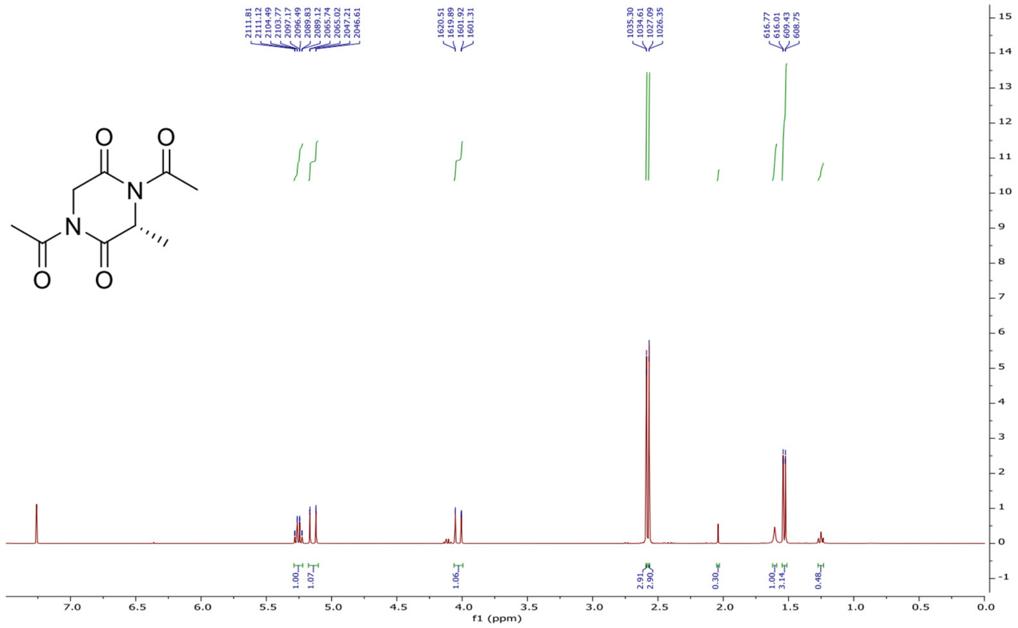

Figure S5

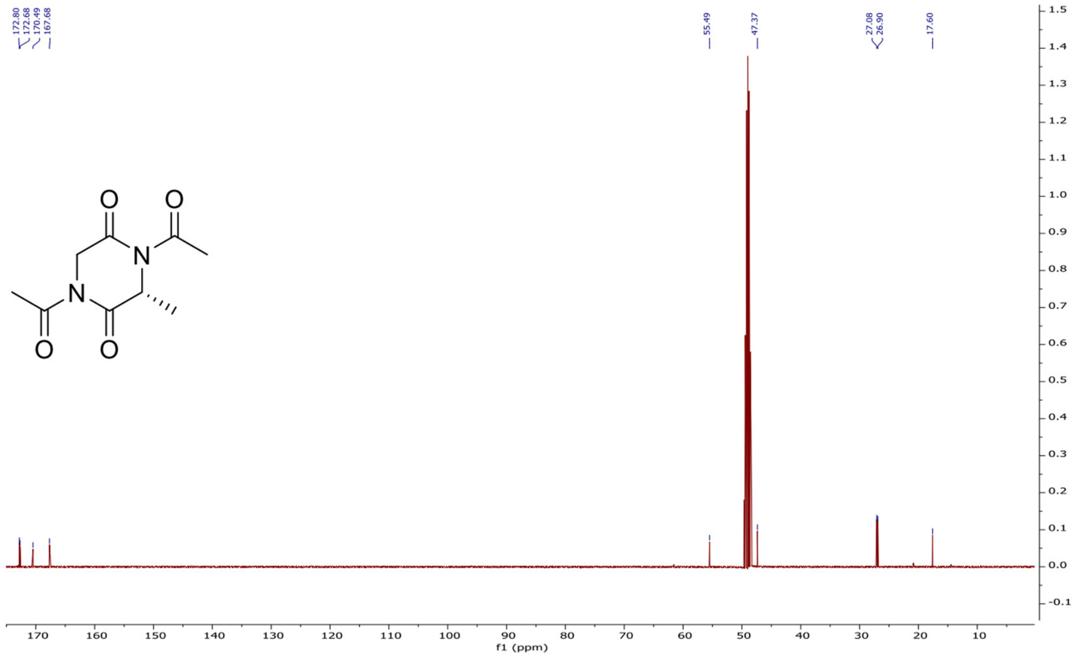

Figure S6

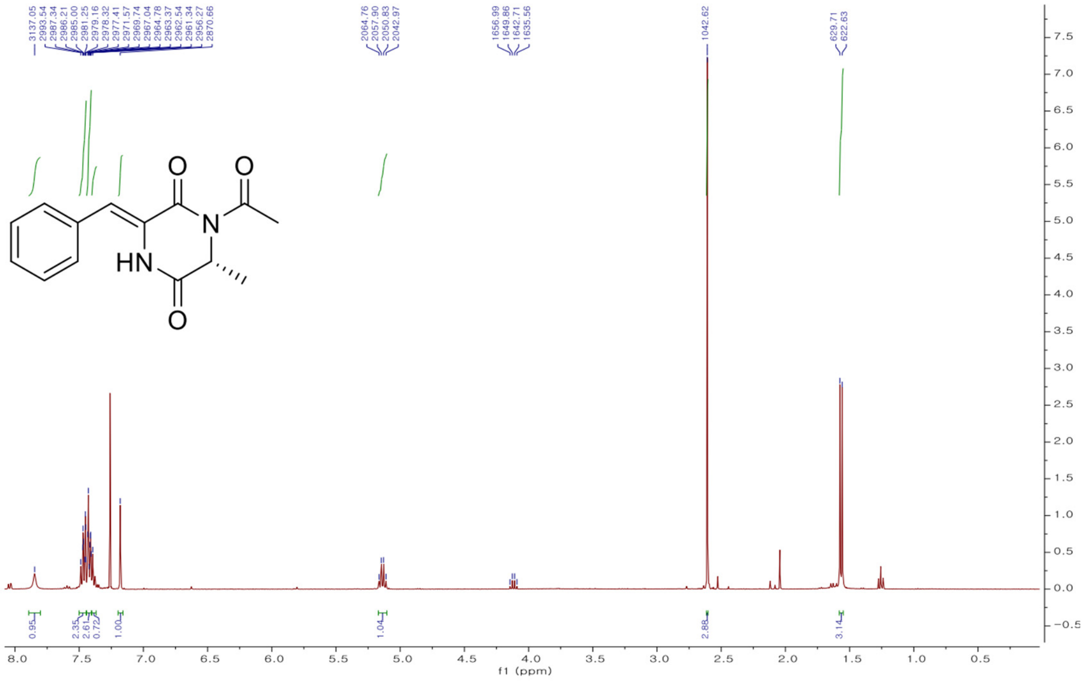

Figure S7

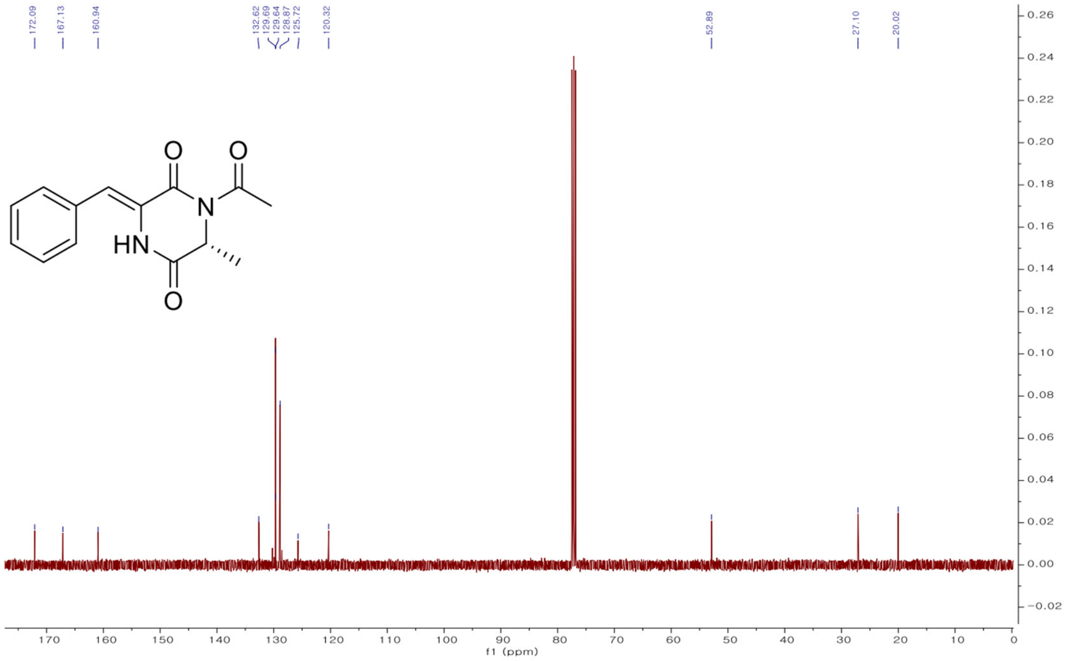

Figure S8

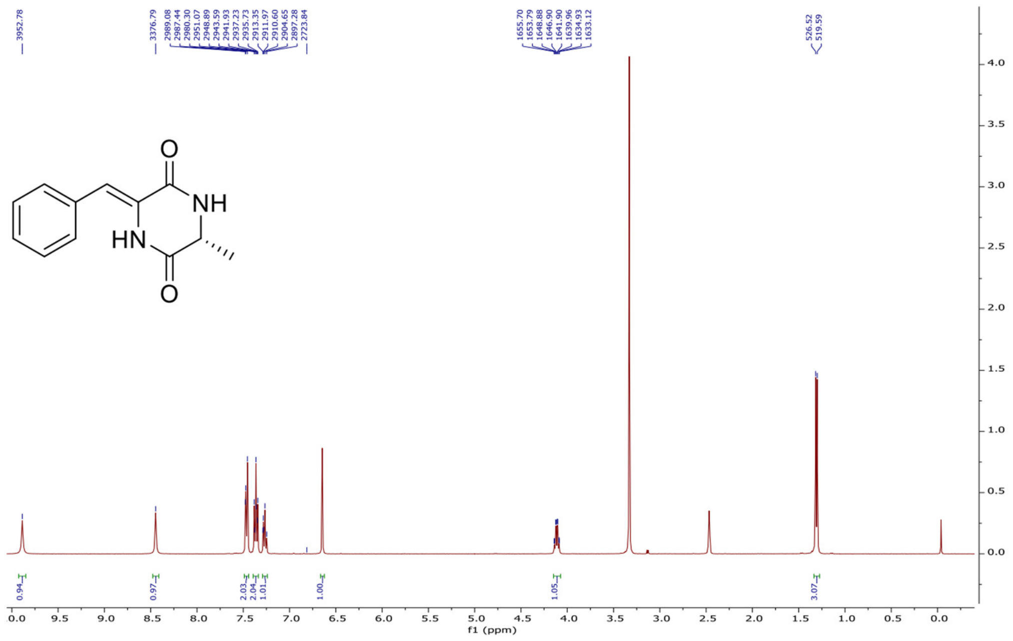

Figure S9

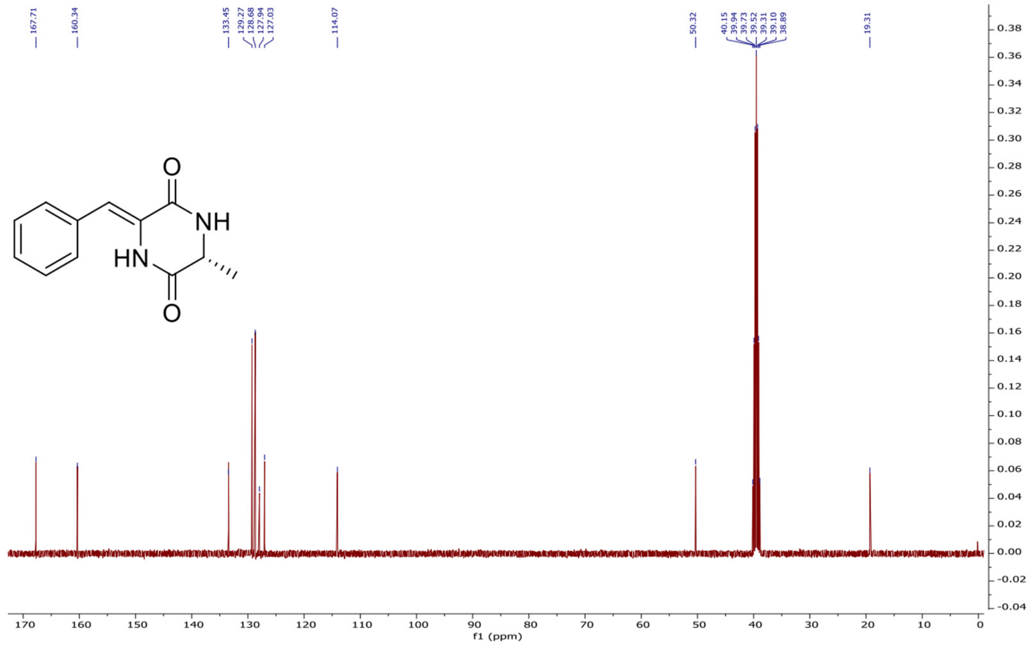

Figure S10

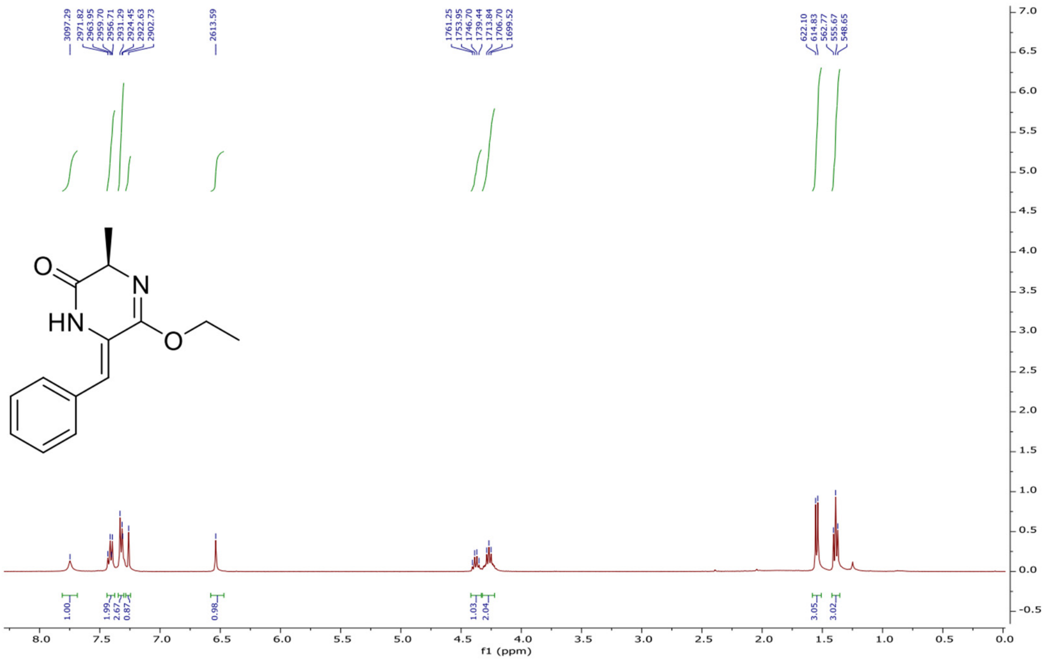

Figure S11

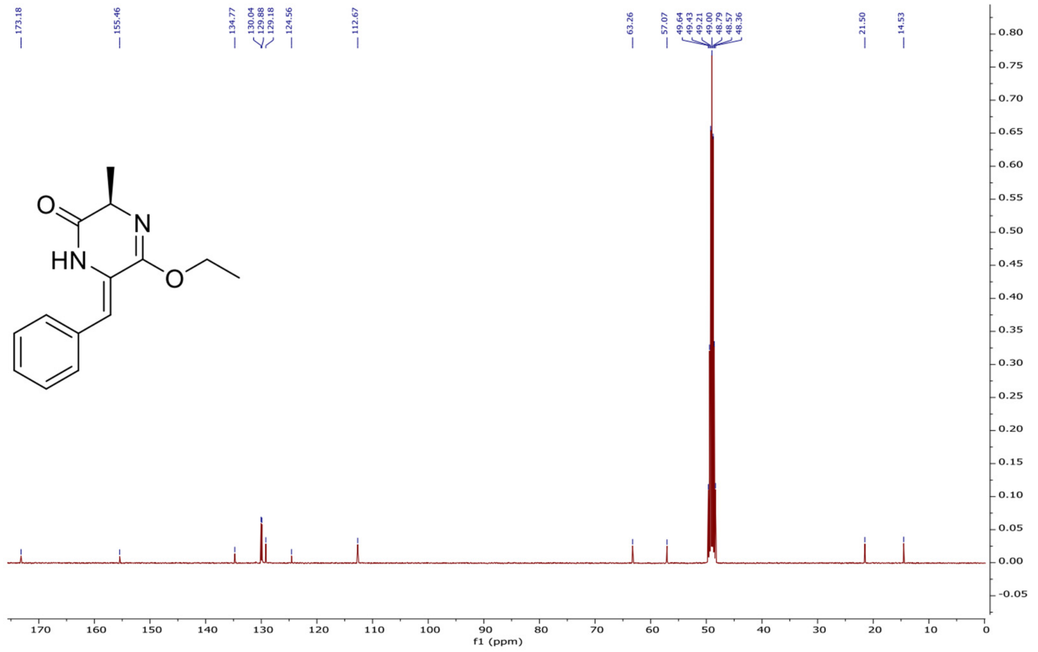

Figure S12

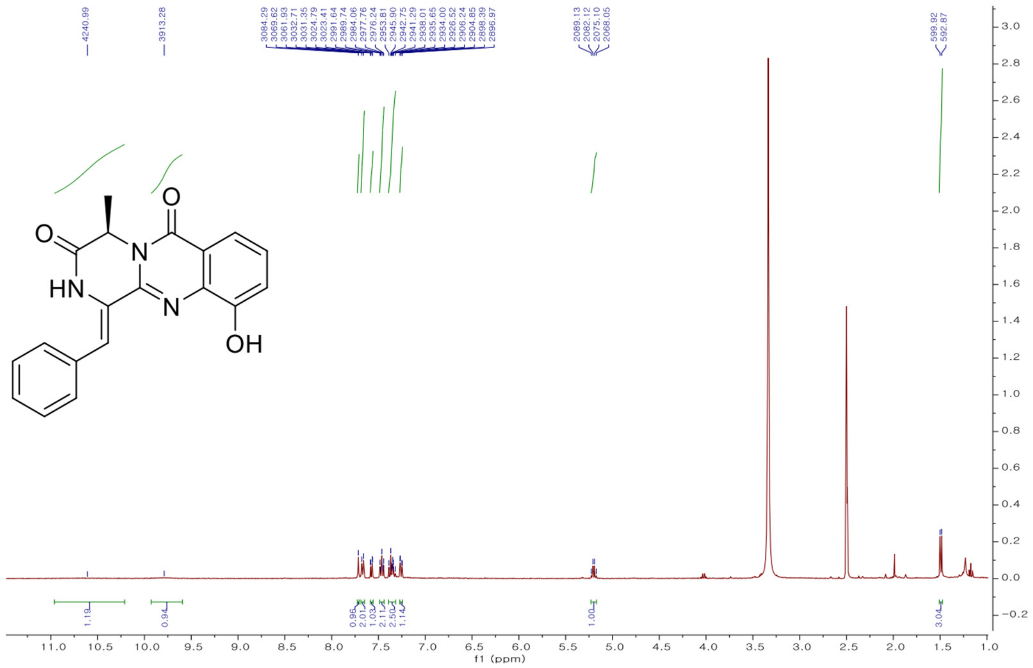

Figure S13

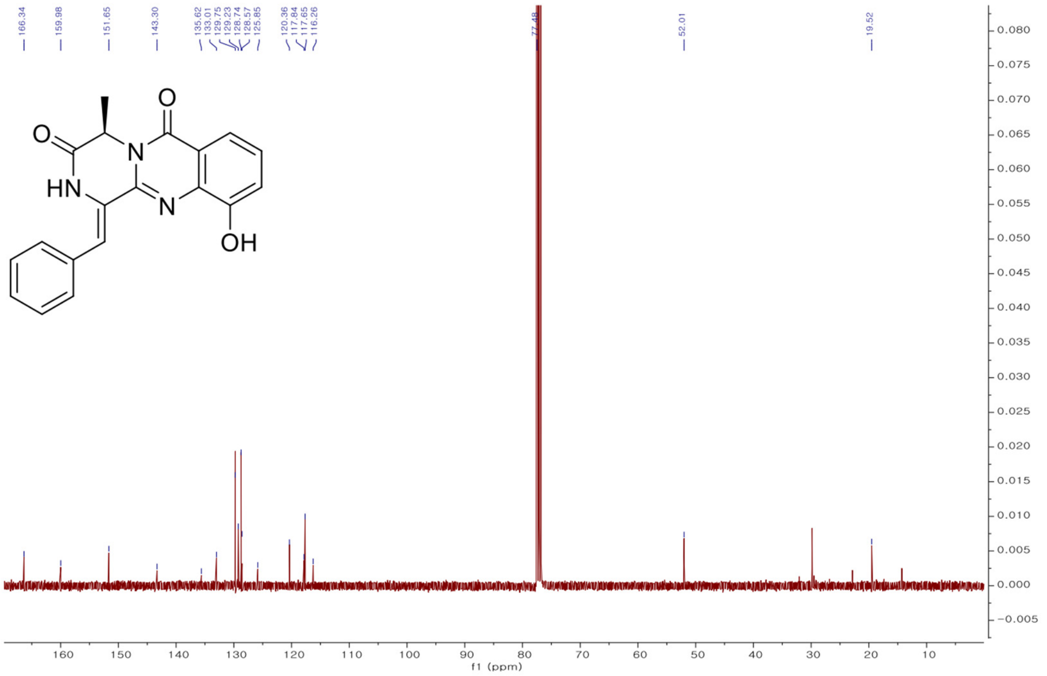

Figure S14

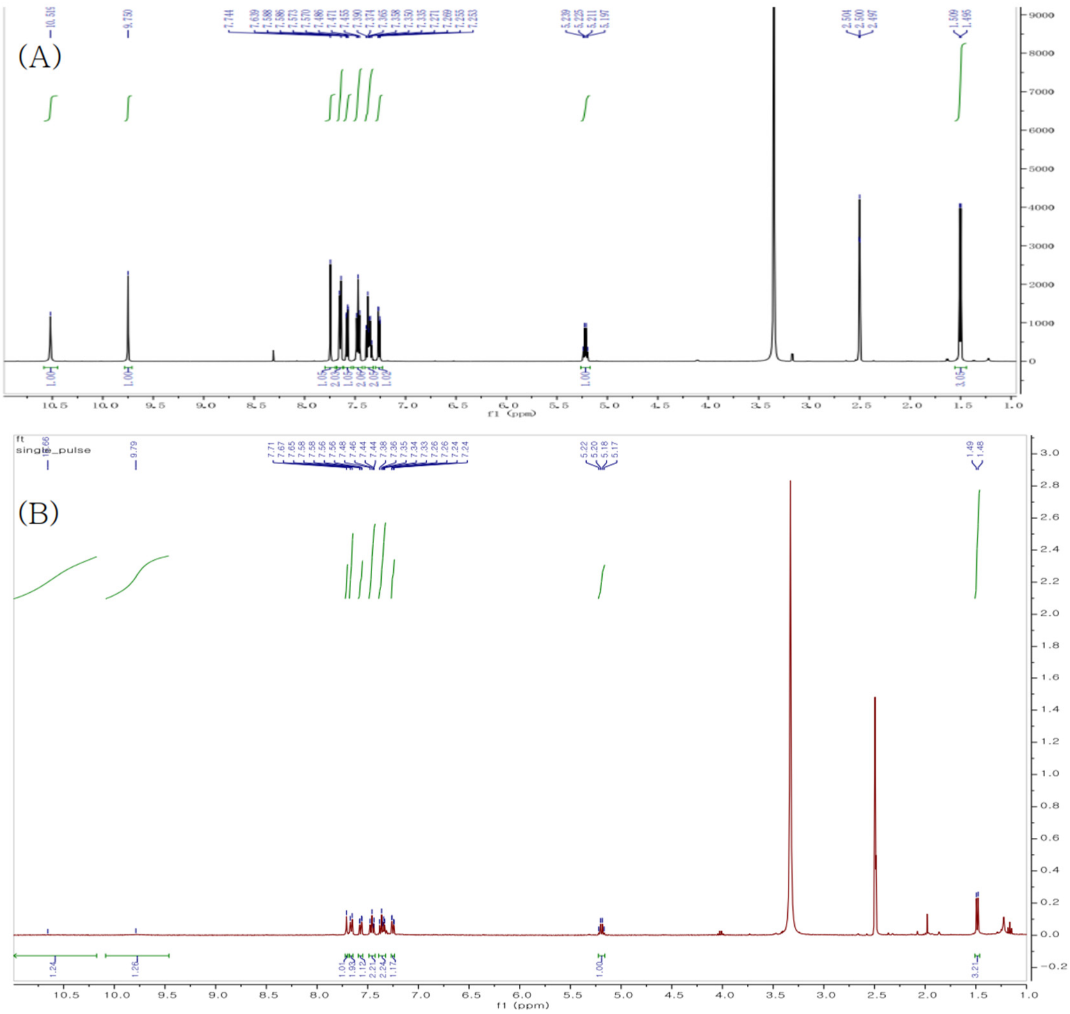

Figure S15
